# Topological Disruption of Structural Brain Networks in Patients With Cognitive Impairment Following Cerebellar Infarction

**DOI:** 10.3389/fneur.2019.00759

**Published:** 2019-07-19

**Authors:** Duohao Wang, Qun Yao, Miao Yu, Chaoyong Xiao, Lin Fan, Xingjian Lin, Donglin Zhu, Minjie Tian, Jingping Shi

**Affiliations:** ^1^Department of Neurology, Affiliated Brain Hospital of Nanjing Medical University, Nanjing, China; ^2^Department of Radiology, Affiliated Brain Hospital of Nanjing Medical University, Nanjing, China; ^3^Department of Neurology, Taizhou People's Hospital, Taizhou, China

**Keywords:** cerebellar infarction, cognitive impairment, diffusion MRI, graph theory, brain network

## Abstract

Cerebellar lesions can lead to a series of cognitive and emotional disorders by influencing cerebral activity via cerebro-cerebellar loops. To explore changes in cognitive function and structural brain networks in patients with posterior cerebellar infarction, we conducted the current study using diffusion-weighted MRI (32 cerebellar infarction patients, 29 controls). Moreover, a series of neuropsychological tests were used to assess the subject's cognitive performance. We found cognitive impairment following cerebellar infarction involving multiple cognitive domains, including memory, executive functions, visuospatial abilities, processing speed and language functions, and brain topological abnormalities, including changes in clustering coefficients, shortest path lengths, global efficiency, local efficiencies, betweenness centrality and nodal efficiencies. Our results indicated that measures of local efficiency, mainly in the precuneus, cingulate gyrus and frontal-temporal cortex, were significantly reduced with posterior cerebellar infarction. At the same time, The correlation analysis suggested thatthe abnormal alterations in the right PCG, bilateral DCG, right PCUN may play a core role in the cognitive impairment following cerebellar infarctions. The differences in topological features of the structural brain networks within the cerebro-cerebellar circuits may provide a new approach to explore the pathophysiological mechanisms of cognitive impairment following cerebellar infarction.

## Introduction

The cerebellum has always been a mysterious structure in the history of neuroscience exploration, especially in the field of advanced cognition. Regarding dysfunction after cerebellar lesions, the main concern in the past has been with motor control and coordination, which mainly manifest as ataxia, balance disorder, vertigo nystagmus, and dysarthria (1). However, recent experimental and clinical evidence suggests that it may also play an important role in cognition and emotion (2–5). The cerebellum cognitive affective syndrome (CCAS), proposed by Schmahmann et al. was characterized by executive dysfunction, spatial cognitive impairment, language deficits, and personality changes (6).

Cerebellar infarction is common in cerebellar lesions, most of which are caused by blockage of the posterior inferior artery. The cerebellar infarction not only affects the patient's motor function and the functional independence but also impacts the patient's cognitive function (7). An increasing amount of evidence has demonstrated that the underlying mechanisms of cognitive impairment following cerebellar lesions might involve abnormal fiber connections between the cerebellum and the functional areas of the cerebrum, especially the frontal and temporal lobes, precuneus and cingulate gyrus (8–10), i.e., the “cerebro-cerebellar loops” (11–14). In particular, the posterior cerebellum is interrelated with the cognitive function areas of the cerebrum (15).

There are many neuroimaging methods for studying brain connections, of which diffusion imaging technology (DTI) is one of the non-invasive methods that can effectively observe and track white matter fiber bundles. It has been applied to the investigation of abnormal brain connections in various cognitive and neuropsychiatric disorders, such as Alzheimer's disease and schizophrenia (16–18). In a previous study, DTI-based fascicular imaging was performed in patients with cerebellar disease to study voxel differences in diffusion coefficient parameters, showing changes in white matter tracts of the cerebellum connecting the cerebellum to cognitive-related cortical areas (19). Furthermore, the relation between cerebellar white matter junction and cognitive impairment was also confirmed in patients with cerebellar neurodegenerative diseases (20). As far as we know, there is currently no DTI study on cognitive impairment after cerebellar infarction.

Whole-brain structural connectivity can be reconstructed with network methods of diffusion MR imaging tractography (21, 22). The present study is the first study of cognitive impairment following cerebellar infarction using the DTI method. The purpose of this study was to investigate topological differences of whole-brain structural connectivity in patients with acute cerebellar infarction and confirm that the cerebellum is involved in cognitive processes by regulating certain cognitive regions of the cerebrum.

## Materials and Methods

### Subjects

The current study recruited 61 subjects from the Affiliated Brain Hospital of Nanjing Medical University from July 2016 to March 2018. All subjects were Chinese and right-handed, including 32 subjects with cerebellar infarction (17 males and 15 females) and 29 age- and sex-matched healthy controls (17 males and 12 females). Eleven patients had right cerebellar infarction, fourteen patients had left cerebellar infarction, and the remaining patients had bilateral cerebellar infarction. The inclusion criteria for the patients with cerebellar infarction were as follows: (1) subjects were aged between 50 and 70 years old; (2) subjects provided informed consent and could cooperate and complete the MRI examination; (3) education period was >5 years, there was no mental retardation and subjects could cooperate and complete the neuropsychological assessment; (4) this was their first cerebellar infarction and the lesion was localized in the posterior cerebellum; and (5) the cerebellar infarction was in the acute phase (the course of disease had been <2 weeks). The exclusion criteria were as follows: (1) Stroke lesions also involve other brain areas besides the posterior cerebellar lobe; (2) could not complete the examination due to various symptoms; (3) previous history of stroke, leukoencephalopathy, brain tumor, brain trauma, encephalitis, metabolic disorders, and other dementia caused by degenerative diseases of the nervous system; (4) mental disorders and intellectual disabilities; and (5) a history of severe heart, brain, or kidney disease or alcohol and drug abuse. In addition, healthy subjects matched by age, sex, and education levels were recruited as healthy controls.

The current study was approved by the Ethics Committee of the Affiliated Brain Hospital of Nanjing Medical University. Each participant had Written informed consent.

### Behavioral Assessment

All participants conducted a series of **behavioral tests** to assess cognitive performance, including episodic memory, working memory, processing speed, executive function, language, and balance function. General mental status was evaluated with the Mini-Mental Status Examination (MMSE) (23). Episodic memory was evaluated with the Rey auditory verbal learning test (RAVLT) (24). Verbal working memory was evaluated with digit span test (DST) (25). Executive function was evaluated with the Trail-Making Test (TMT) (26) and verbal fluency task (VFT) (27). Language function was evaluated using the Boston naming test (BNT) (28). Visuospatial abilities was evaluated with the clock drawing test (CDT) (29). To assess the emotional state of the patient, we used the Hamilton Depression Scale(HAMD) (30). The assessment of balance function used the Berg balance scale (BBS) (31) and International Cooperative Ataxia Rating Scale(ICARS) (32). Behavioral assessment were conducted by psychometricians and psychologists.

### MRI Data Acquisition

The subjects underwent magnetic resonance scans using Siemens 3.0T scanner (Erlangen, Germany) with standard orthogonal head coils. For all subjects, Conventional T2-weighted MR images were obtained to rule out cortical atrophy and other brain abnormalities: repetition time (TR) = 3,500 ms; echo time (TE) = 103 ms; flip angle = 90°; thickness = 6.0 mm; gap = 0 mm; acquisition matrix = 320 × 192; field of view (FOV) = 240 × 240 mm^2^; number of excitations (NEX) = 2. the high resolution T1-weighted image were collected in a sagittal direction using a 3D-SPGR sequence. The T1 weighted image parameters were as follows: 176 sagittal slices; 1.0 mm slice thickness; TR = 1,900 ms; TE = 2.48 ms; flip angle = 9°; matrix = 512 × 512. The diffusion-weighted MR image were obtained using a spin echo planar imaging sequence, the parameters of which were as follows : TR = 6,600 ms; TE = 93 ms; 45 axial slices; slice thickness = 3.0 mm;gap = 0 mm; 30 gradient directions with a *b*-value = 1,000 sec/mm^2^; acquisition matrix = 128 × 128; FOV = 240 × 240 mm^2^. Imaging was performed by a physician with 16 years of experience in MR image acquisition.

### Data Preprocessing

The data preprocessing steps were mainly as follows: Eddy current and head movement correction of the diffusion-weighted image data were conducted by applying affine correspondence of DTI to the image of b = 0. Excess scalp and brain tissue were removed, and then the three eigenvalues (λ1, λ2, λ3) and the eigenvectors were obtained by diagonalizing the tensor matrix. The fractional anisotropy (FA) were calculated. At the same time, the matrix, and inverse matrix from the individual space to the standard space were calculated. The AAL template was used to divide the cerebral cortex and the subcortical region into 90 brain regions, with each brain region as a network node (33). The AAL template by inverse matrix registration to the individual brain space completes the definition of individual brain network nodes. The AAL standard image under the individual spatial template was constructed with the FA image, and the fiber network was constructed to generate a 90 × 90 matrix of the average FA value of all the voxels along the fiber bundle. The above processing were completed by PANDA software (http://www.nitrc.org/projects/panda). The characteristic parameters of the brain network were constructed by Gretna software (http://www.nitrc.org/frs/download.php/5534/gretna.zip). Finally, BrainNet Viewer (http://www.nitrc.org/projects/bnv/) was used to render the multidimensional visualization of weighted networks. It is worth noting that in the construction of the brain network, we defined a threshold for the definition of the network edges, that is, if the number of streamlines between two nodes were greater than a certain threshold (T = 3), then there was an edge connection between the two regions. This threshold was used in previous network research because of its advantages that can reduce the false positive rate of network connection caused by noise or limitation in the study of deterministic tractography (34, 35).

### Topological Characteristics

Graph theory is a graph composed of several nodes and edges, which is usually used to describe the connection between two nodes. In the brain networks, nodes and edges represent brain regions and connections between two regions, respectively. Graph theoretical analyses have revealed that brain networks have important topological parameters, the most striking of which was the small worldness (36). In the study of structural networks, several key parameters were used, including local efficiency (*Eloc*), global efficiency (*Eglob*), clustering coefficient (*Cp*), normalized clustering coefficient (γ), shortest path length(*Lp*), normalized characteristic path length (λ), small-worldness (σ), betweenness centrality, and regional efficiency.

The clustering coefficient C(i) of node i represents the ratio of the number of edges where the actual number of edges E(i) is completely connected to the subgraph Gi. Gi is the connection subgraph of all nodes connected to node i, and Ki is the degree of node i. The formula is as follows:

$$\begin{array}{l} \text{C}\left(\text{i}\right)=\frac{2\text{E}}{\left(\text{i}\right)\text{Ki}\left(\text{Ki}-1\right)}. \end{array}$$

The clustering coefficient *Cp* of the entire network is defined as the average of the clustering coefficients of all nodes in the network and reflects the connection status of the entire network. The formula is as follows:

$$\begin{array}{l} Cp=1/N\sum_{i=1}^{N}C\left(i\right). \end{array}$$

The shortest path length is indicated by L, and the shortest distance Lij from node i to node j refers to how many times the connection from node i can reach node j. The average of the distances of all nodes is the average shortest path length of the entire network. The shortest path length (*Lp*) measures the extent of network long-distance connections. The formula is as follows:

$$\begin{array}{l} Lp\left(G\right)=\frac{1}{N}\sum_{i\epsilon G}Li. \end{array}$$

Global efficiency (*Eglob*) measures the global efficiency of the information transmit in the network. The global efficiency is defined as the average of the reciprocal of the shortest path of all nodes in the network. The formula is as follows:

$$\begin{array}{l} E_{glob}\left(G\right)=\frac{1}{N\left(N-1\right)}\sum_{i\neq j\epsilon G}1/Lij. \end{array}$$

The local efficiency(Eloc) is defined as the average of the inverse of the shortest path of all nodes in the subgraph Gi. The formula is as follows:

$$\begin{array}{l} E_{loc}\left(G\right)=\frac{1}{N}\sum_{i\epsilon G}\text{Eglob }\left(\text{Gi}\right). \end{array}$$

Regional efficiency, *E*nodal(*i*) is the nodal properties of the structural networks, which indicates the average shortest path length between node i and all other nodes in the structural network. The *E*nodal(*i*), is defined as follows:

$$\begin{array}{l} E_{nodal}\left(i\right)=\frac{1}{N-1}\sum_{i\neq j\epsilon G}1/Lij. \end{array}$$

Betweenness centrality of each node can be used to define the hub regions of the brain network (37). The centrality level of the node indicates the importance of node i in promoting connection between different regions of the brain network. The definition of the hub can be implemented in a variety of ways, such as node efficiency, betweenness centrality and nodal degree (37). The definition of the hub regions in the current study was based on betweenness centrality. If the centrality value of node i was >1.5 times the average centrality of the brain network, the node was identified as a hub region in the brain network.

Small-worldness network characteristics were proposed by Watts and Strogatz (38), including γ, λ, σ. The λ is defined by λ = ${\text{L}}_{p}^{real}$*/*${\text{L}}_{p}^{rand}$ and the γ is defined by γ = ${\text{C}}_{p}^{real}$*/*${\text{C}}_{p}^{rand}$. Small-worldness, σ = γ*/*λ, is typically > 1 for small-world networks. Compared with random networks, the advantage of small-world networks is that it has higher interconnectivity and similar shortest path length.

### Statistical Analysis

Using SPSS 20.0 statistical software, two-sample *T*-test was used to compare demographic and clinical data on the basis of the normal distribution test. The age, education level and neuropsychological test scores of the cerebellar infarction group and the control group were compared by independent two-sample *t*-tests. The sex distribution of the two groups were compared by chi-square test. Network parameters of the two groups were compared by an independent two-sample *t*-test. The false discovery rate correction was used to correct the statistical results of the network hubs and regional efficiency. Pearson correlation analyses were calculated between the differences in whole brain structural connectivity and neuropsychological test scores; among them, we only examined network characteristics with significant intergroup differences. The significance threshold in statistical analyses were *P* < 0.05.

## Results

### Demographics and Clinical Results

The demographic data and **clinical** characterizations for each group are shown in Table 1. There were no significant differences in age, sex, and educational level between the cerebellar infarction group and the control group. There were significant differences in MMSE scores (*P* < 0.001) between the two groups. For the domain of episodic memory, working memory, executive function, visuospatial abilities, language and balance function, the group effects were significant for AVLT (*P* < 0.01), DST (*P* < 0.05), TMT (TMT-A: *P* < 0.01), TMT-B (*P* < 0.001), VFT (*P* < 0.001), CDT(*P* < 0.05), BNT (*P* < 0.01), BBS (*P* < 0.001) and ICARS(*P* < 0.001). There was no significant difference in the HAMD scores between the two groups.

**Table 1:** Demographic and clinical characteristics.

**Demographic and clinical data**	**Cerebellar infarction(*n* = 32)**	**Control group (*n* = 29)**	***P*-value**
Sex (M/F)	17/15	17/12	0.799
Age (year)	61.28 ± 6.45	61.00 ± 4.71	0.848^#^
Education level (year)	8.03 ± 1.47	8.38 ± 1.21	0.319
MMSE	25.00 ± 2.88	28.07 ± 1.33	<0.001
RAVLT-A	21.13 ± 3.68	23.52 ± 2.81	0.006
RAVLT-B	2.78 ± 1.16	4.79 ± 0.98	<0.001
DST	10.84 ± 2.46	12.34 ± 2.00	0.012
BNT	23.16 ± 3.82	26.00 ± 2.07	0.001
TMT-A	105.41 ± 40.91	64.93 ± 12.58	<0.001
TMT-B	265.72 ± 85.62	163.89 ± 49.00	<0.001
VFT	23.63 ± 4.55	29.52 ± 4.52	<0.001
CDT	3.91 ± 0.99	4.88 ± 0.74	0.013
HAMD	0.16 ± 0.45	0.00 ± 0.00	0.065
BBS	51.22 ± 4.66	56.00 ± 0.00	<0.001
ICARS	6.31 ± 3.53	0.00 ± 0.00	0.000

*MMSE, Mini-Mental State Examination; RAVLT, Rey auditory verbal learning test; DST, digit span test; BNT, Boston naming test; TMT, Trail-Making Test; VFT, verbal fluency task; CDT, Clock drawing test; HAMD, Hamilton Depression Scale; BBS, Berg Balance Scale; ICARS, International Cooperative Ataxia Rating Scale. Values are represented as the mean ± SD, #p-value for the sex distribution in the two groups was obtained using a χ2-test, other using independent-samples t-test, p <0.05 was considered significant*.

### Structural Network Parameters

**Figure 1:**
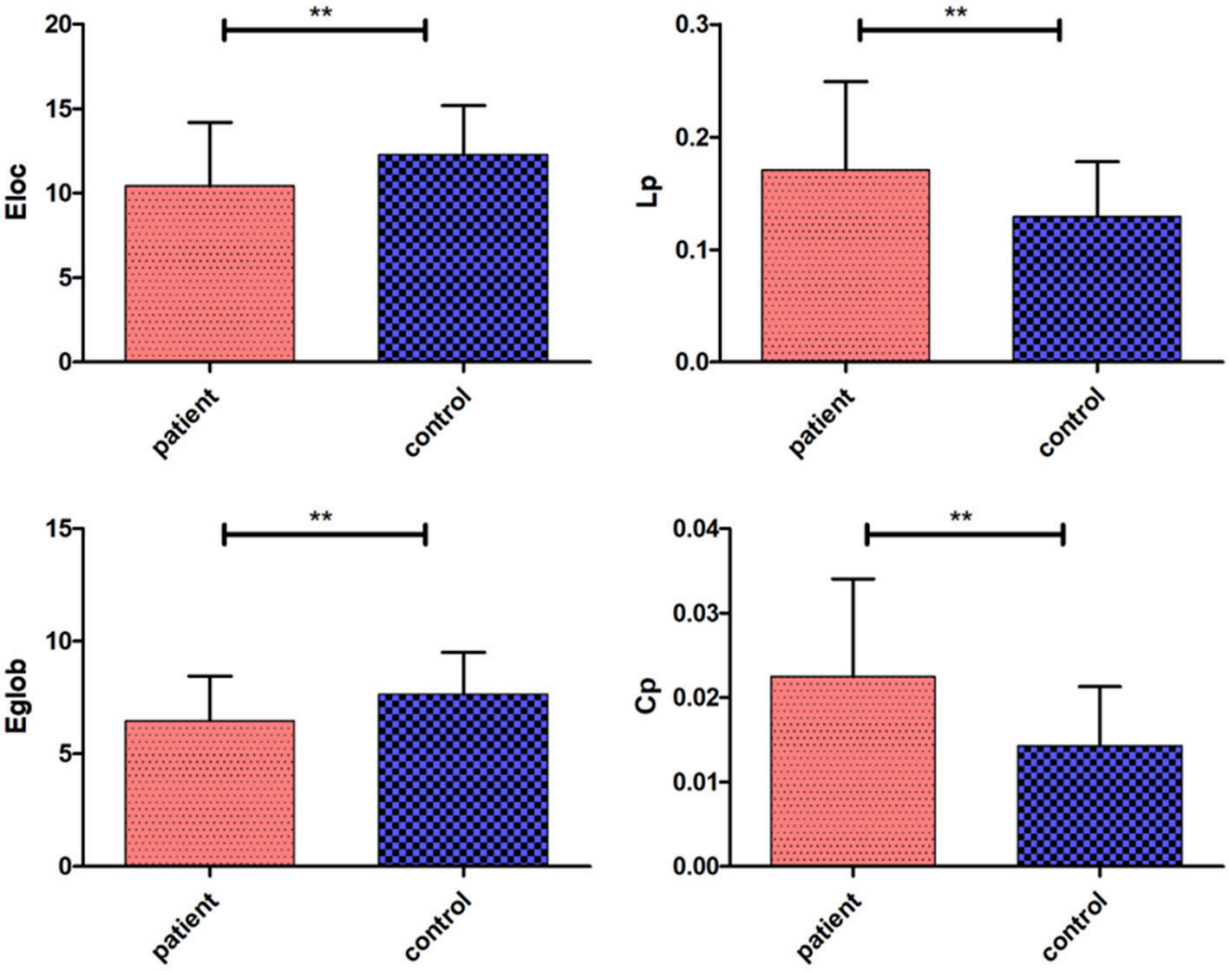
Significantly different network parameters at a threshold T = 3 between cerebellar infarction and healthy controls. Bars and error bars indicate the mean and standard deviation. The asterisk marks indicate a significant difference between the two groups (*p* < 0.05).

There were significant differences in the network parameters *Eglob, Eloc, Cp*, and *Lp* between the cerebellar infarction group and the control group. The cerebellar infarction group was found to have decreased parameters of global efficiency and local efficiency compared with the control group. In contrast, the clustering coefficient and shortest path length increased in patients with cerebellar infarction (Table 2, Figure 1). Other parameters, such as the normalized clustering coefficient, normalized characteristic path length and small-worldness, did not show significant differences between the groups.

**Table 2:** Structural network parameters in the cerebellar infarction and control group.

**Parameters of network**	**Cerebellar infarction (*n* = 32)**	**Control group (*n* = 29)**	***T*-value**	***P*-value**
*Eglob*	6.61 ± 2.34	7.91 ± 2.18	−2.23	0.030^*^
*Eloc*	10.43 ± 3.37	12.27 ± 2.92	−2.12	0.038^*^
*Cp*	2.29 ± 1.16^&^	1.51 ± 0.61^&^	3.24	0.002^*^
γ	5.00 ± 0.82	4.70 ± 0.70	1.52	0.135
λ	1.15 ± 0.06	1.12 ± 0.06	1.63	0.108
*Lp*	1.72 ± 0.69^#^	1.37 ± 0.42^#^	2.37	0.021^*^
σ	4.36 ± 0.67	4.19 ± 0.62	1.03	0.309

*There were significant differences in the network parameters Eglob, Eloc, Cp and Lp between the cerebellar infarction group and the control group. Eglob, global efficiency; Eloc, local efficiency; Cp, clustering coefficient; γ, normalized clustering coefficient; λ, normalized characteristic path length; Lp, shortest path length, σ, small-worldness. Values are represented as the mean ± SD*.

& indicate *10^−2^

# indicate *10^−1^.

* *P <0.05 was considered significant*.

### Network Hubs

**Figure 2:**
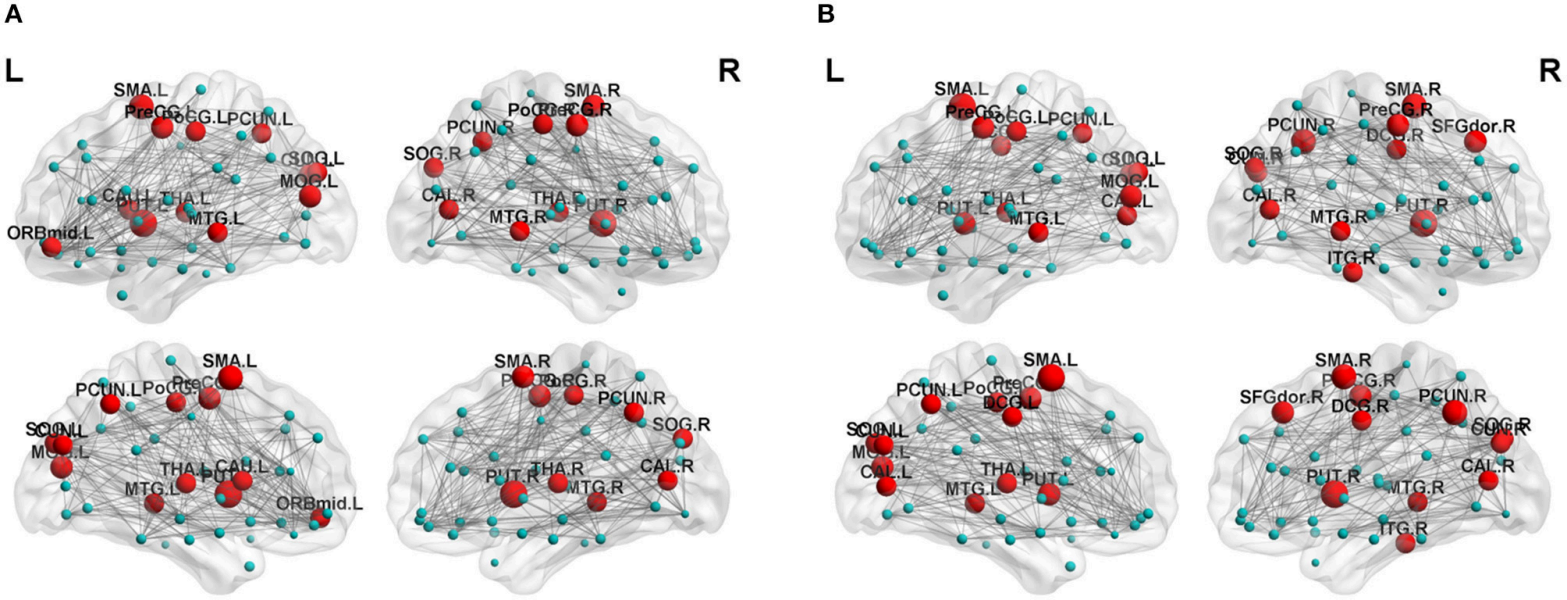
Distribution of hubs in the cerebellar infarction group **(A)** and control group **(B)**. The hub nodes are shown in red, and node sizes represent their betweenness centrality values. For abbreviations of the hub regions, please see Table 2.

**Figure 3:**
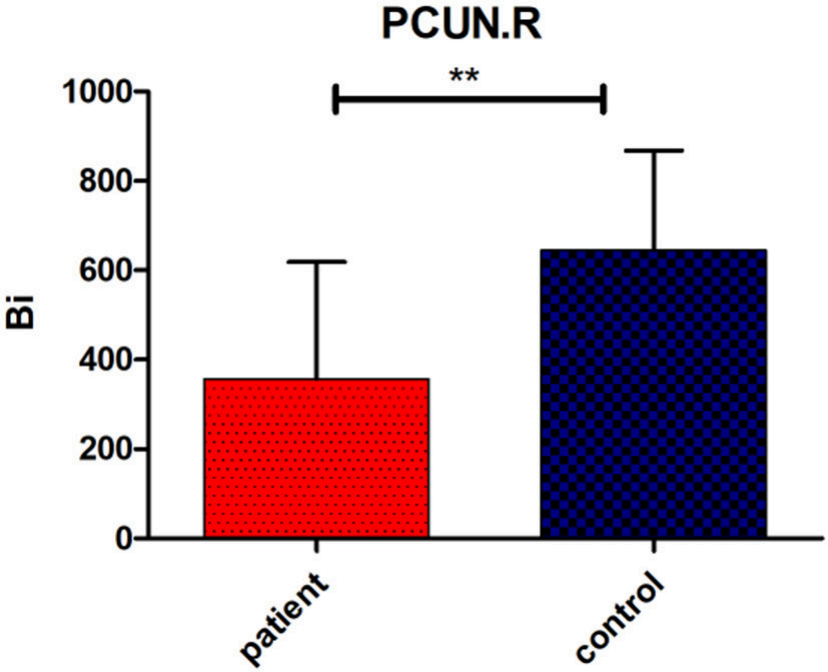
Significant group difference in betweenness centrality value (Bi) of right precuneus (PCUN.R). Bars and error bars indicate the mean and standard deviation. The asterisk marks indicates a significant difference between the two groups (*p* < 0.05; false discovery rate corrected).

Regarding brain networks, regions with high levels of centrality can be identified as network hubs. The definition of hub regions in the current study was based on betweenness centrality. If the betweenness centrality value of node was >1.5 times the average centrality of the network, the node was identified as a hub region in the brain network (37). The hub distributions of patients and controls are shown in Table 3 and Figure 2. There were 21 hub regions in the cerebellar infarction group and 22 in the control group. Among them, five hubs only existed in the patient group, including the right thalamus (THA.R), left middle temporal gyrus (MTG.L), left caudate nucleus (CAU.L), left middle frontal gyrus, orbital part (ORBmid.L), and right postcentral gyrus (PoCG.R). In contrast, the right inferior temporal gyrus (ITG.R), bilateral median cingulate and paracingulate gyri (DCG), right cuneus (CUN.R), right superior frontal gyrus, dorsolateral (SFGdor.R), and left calcarine fissure and surrounding cortex (CAL.L) were defined as hub regions only in the control group. Among all hub regions, the most prominent hub of the two groups was the right lenticular nucleus, putamen (PUT.R). Subsequently, the hubs in both groups were compared, and it was found that there was a significant difference only in the right precuneuses (PCUN.R) (*P* < 0.01; false discovery rate corrected). The details are shown in Figure 3.

**Table 3:** Hub regions in the cerebellar infarction and control group.

	**Hub region**	**Mean of Bi**		**Hub region**	**Mean of Bi**
Patients	PUT.R	915	Controls	PUT.R	842
	PUT.L	869		SMA.L	742
	SMA.L	635		SMA.R	679
	SMA.R	531		PCUN.R	661
	MOG.L	531		PUT.L	645
	PreCG.L	511		MOG.L	548
	PreCG.R	463		PreCG.L	490
	SOG.L	415		PreCG.R	441
	PCUN.L	390		SFGdor.R	422
	PCUN.R	369		PCUN.L	390
	PoCG.L	362		SOG.L	371
	THA.R	356		CAL.R	351
	MTG.R	339		DCG.R	346
	ORBmid.L	334		CUN.R	337
	SOG.R	331		THA.L	321
	MTG.L	325		MTG.R	304
	CAL.R	304		CAL.L	303
	THA.L	292		CUN.L	296
	PoCG.R	278		SOG.R	288
	CAU.L	275		PoCG.L	279
	CUN.L	271		DCG.L	266
				ITG.R	265

*If the betweenness centrality value of the node was >1.5 times the average centrality of the network, the node was identified as a hub region in the brain network. There were 21 hub regions in the cerebellar infarction group and 22 in the control group. Sixteen hub regions of the two groups were the same, 5 were only present in the cerebellar infarction group, and 6 were only present in the control group. PUT, lenticular nucleus, putamen; SMA, supplementary motor area; MOG, middle occipital gyrus; PreCG, precentral gyrus; SOG, superior occipital gyrus; PCUN, precuneus; PoCG, postcentral gyrus; THA, thalamus; MTG, middle temporal gyrus; ORBmid, middle frontal gyrus, orbital part; CAL, calcarine fissure and surrounding cortex; CAU, caudate nucleus; CUN, cuneus; SFGdor, superior frontal gyrus, dorsolateral; DCG, median cingulate and paracingulate gyri; ITG, inferior temporal gyrus*.

### Regional Efficiency

**Figure 4:**
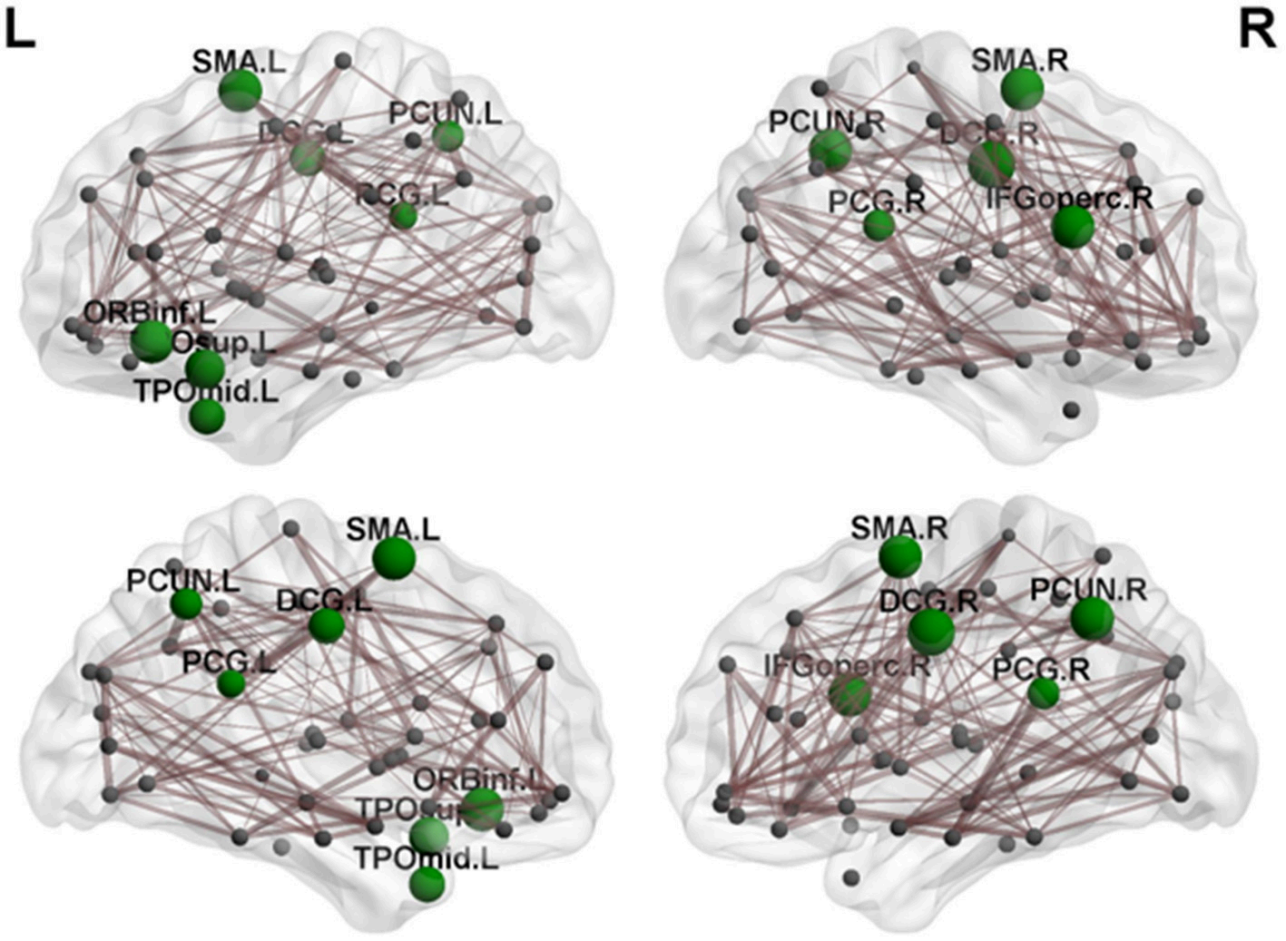
By comparing nodal efficiency between the cerebellar infarction and control group, 12 brain regions with significant differences (*P* < 0.05, false discovery rate corrected) between the two groups are shown in green. Node sizes represent the significance of between-group differences in regional nodal efficiency. For the abbreviations of the regions, please see Table 3.

Regional efficiency in the cerebellar infarction group was lower than the control group (Table 4, Figure 4). Twelve brain regions with significant differences (*P* < 0.05; false discovery rate corrected) between the two groups were found, mainly distributed in the precuneus, cingulate gyrus and frontal-temporal cortex, including bilateral median cingulate and paracingulate gyri (DCG), right inferior frontal gyrus, opercular part (IFGoperc.R), left inferior frontal gyrus, orbital part (ORBinf.L), bilateral supplementary motor area (SMA), bilateral precuneus (PCUN), left temporal pole, superior temporal gyrus (TPOsup.L), left temporal pole, middle temporal gyrus (TPOmid.L), and bilateral posterior cingulate gyrus (PCG).

**Table 4:** Brain regions with significant differences in nodal efficiency among the two groups.

**Region**	***T*-value**	***P*-value**
DCG.R	−4.054	0.000
IFGoperc.R	−3.632	0.001
ORBinf.L	−3.632	0.001
SMA.L	−3.559	0.001
SMA.R	−3.456	0.001
PCUN.R	−3.389	0.001
TPOsup.L	−3.258	0.002
DCG.L	−3.116	0.003
TPOmid.L	−3.101	0.003
PCG.R	−2.995	0.004
PCUN.L	−2.965	0.004
PCG.L	−2.926	0.005

*Nodal efficiency was compared between the two groups using an independent-samples t-test. P <0.05 (false discovery rate corrected) was considered to be significantly different between the two groups. For T values, each negative column represents cerebellar infarction group < the control group. DCG, median cingulate and paracingulate gyri; IFGoperc, inferior frontal gyrus, opercular part; ORBinf, inferior frontal gyrus, orbital part; SMA, supplementary motor area; PCUN, precuneus; TPOsup, temporal pole, superior temporal gyrus; TPOmid, temporal pole, middle temporal gyrus; PCG, posterior cingulate gyrus*.

### Relationship Between Neuropsychological Scale Scores and Brain Network Characteristics

**Figure 5:**
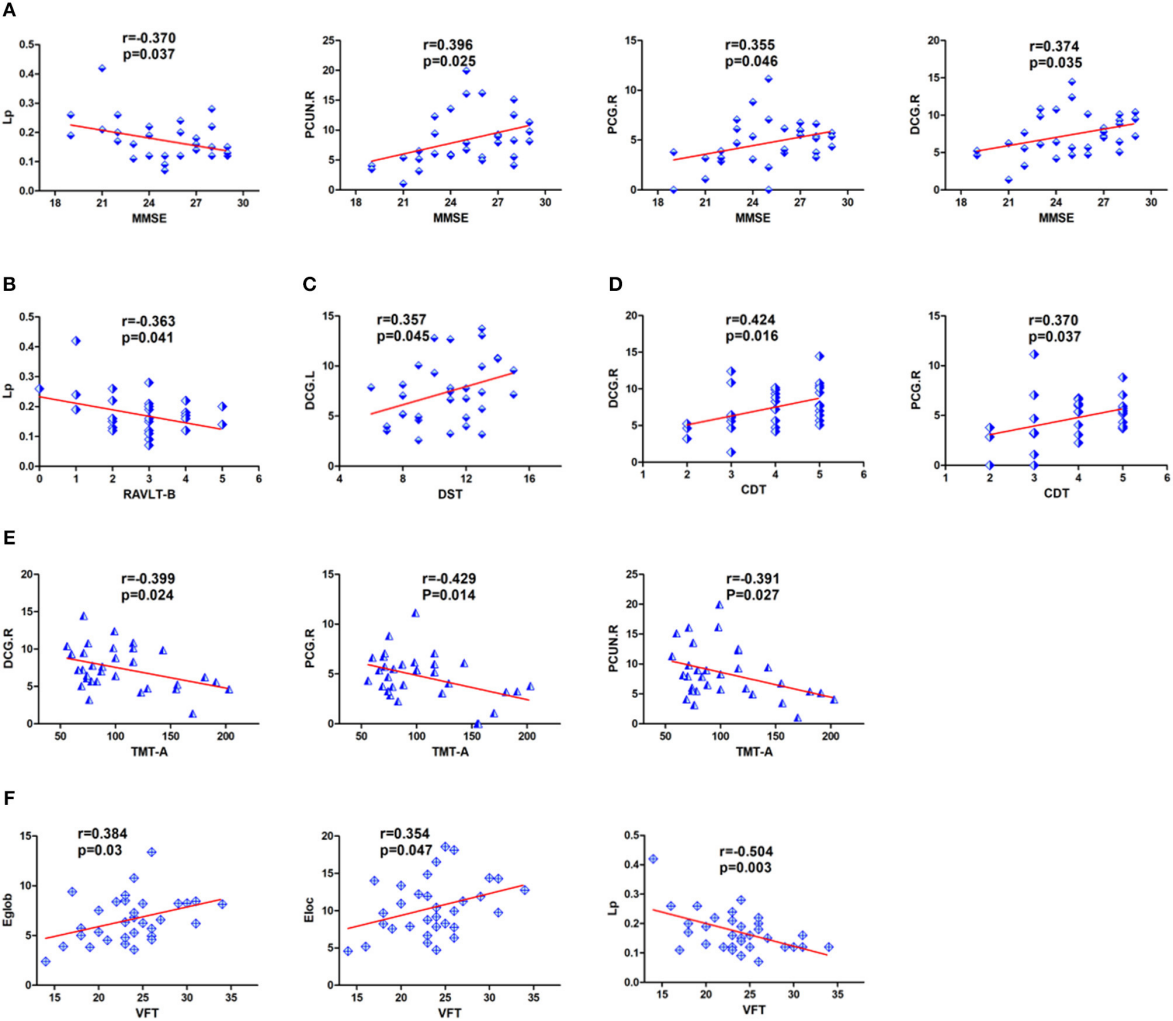
Detailed display of the correlations between neuropsychological scores and regional nodal efficiency and network parameters in patients with cerebral infarction. **(A)** MMSE was significantly correlated with nodal efficiency in DCG.R, PCUN.R, PCG.R and *Lp*. **(B)** RAVLT-B showed a significant negative correlation with *Lp*. **(C)** DST showed a significant positive correlation with nodal efficiency in DCG.L. **(D)** CDT scores were positively correlated with the nodal efficiency of DCG.R and PCG.R. **(E)** TMT-A showed a significant negative correlation with nodal efficiency in DCG.R, PCG.R, and PCUN.R. **(F)** VFT showed a significant correlation with *Eloc, Lp and Eglob*. For the abbreviations of nodes and neuropsychological scores, see Tables 1, 3.

Pearson correlation analyses were calculated between the differences in whole brain structural connectivity and neuropsychological test scores. Among them, we only examined network characteristics with significant intergroup differences. MMSE scores were significantly negatively correlated with *Lp* (*r* = −0.370, *P* = 0.037) and significantly positively correlated with the nodal efficiency of DCG.R (*r* = 0.374, *P* = 0.035), PCG.R (*r* = 0.355, *P* = 0.046) and PCUN.R (*r* = 0.396, *p* = 0.025) (Figure 5A). RAVLT-B scores showed a correlation with *Lp* (*r* = −0.363, *P* = 0.041) (Figure 5B). DST scores were positively correlated with the nodal efficiency of DCG.L (*r* = 0.357, *p* = 0.045) (Figure 5C). CDT scores were positively correlated with the nodal efficiency of DCG.R (*r* = 0.424, *p* = 0.016) and PCG.R(*r* = 0.370, *P* = 0.037) (Figure 5D). TMT-A performance was negatively correlated with the nodal efficiency of PCG.R (*r* = −0.429, *p* = 0.014), DCG.R (*r* = −0.399, *P* = 0.024), and PCUN.R (*r* = −0.391, *p* = 0.027) (Figure 5E). VFT scores were significantly positively correlated with *Eloc* (*r* = 0.354, *P* = 0.047) and *Eglob* (*r* = 0.384, *P* = 0.03) and negatively correlated with *L*p (*r* = −0.504, *P* = 0.003) (Figure 5F).

## Discussion

To the best of our knowledge, there have been no previous studies using DTI methods to investigate the correlations between cerebellar and cognitive functions in patients with cerebellar infarctions. In this study, we found that cognitive impairment after cerebellar infarction was involved in multiple cognitive domains and the topological alterations of brain networks, including *Cp, Lp, Eglob, Eloc*, betweenness centrality and nodal efficiency. Additionally, we studied the correlation between the differences in whole brain structural connectivity and neuropsychological scores, which suggested that brain topological abnormalities may have played an important role in the cognitive impairment following cerebellar infarctions.

In our study, *Cp* and *Lp* in the cerebellar infarction group were increased significantly compared to those measures in the control group, while *Eglob* and *Eloc* were decreased in the patient group compared to the control group. Decreased *Eglob* and *Eloc* indicated that brain network efficiency was diminished and integration of the brain networks was disrupted in patients with cerebellar infarction. Current research has also suggested that the cerebellum regulates the brain's higher-order cognitive processes by forming a wide range of neural circuits with specific brain regions (39). Meanwhile, many previous studies have shown that brain networks have small-world properties (40, 41). The small-world networks have quantified the balance between separation and integration of processing information and have enabled the brain to process information efficiently. Previous research on brain networks showed that small-world networks have both higher *Cp* and shorter *Lp* (38). Increased *Lp* and decreased *Cp* suggested a reduction in the global integration of the structural brain, i.e., destruction of small-world properties (42). Our study did not find abnormalities in the small-world properties, including γ, λ, σ. However, we found that *Cp* and *L*p were significantly increased in the cerebellar infarction group. Increased *Lp* indicated that the integration of the brain networks was disrupted, which was consistent with previous research results. These measures may suggest a potential change in the small-world properties after cerebellar infarction. There is currently no reasonable explanation for the increased *Cp*. It may reflect the earliest adaptive mechanism in the brain network disruption, which requires further confirmation by future large-sample trials.

Previous research on brain networks has shown that hub regions were the core nodes in the structural network of the brain (35). The centrality plays an important role in global information transmission. The present study found that the distribution of hub regions in the cerebellar infarction group was similar with control group. Only a few central distributions were different between the two groups, which may indicate the changes of brain structure network after cerebellar infarction. Among all hub regions, the most prominent hub of the two groups was the right lenticular nucleus, putamen (PUT.R). The proposed brain region is an essential part of extrapyramidal system, which is consistent with the previous report that the important function of cerebellum is motor assistance and coordination. Among the hub regions in both groups, only the right precuneus showed a significant difference between the two groups. In a prior study, Dicks et al. used structural magnetic resonance imaging to study patients with mild cognitive impairment and found that the betweeness centrality value of the precuneus was significantly correlated with cognitive decline (43). In our exploration, the betweeness centrality value of precuneus in patients with cerebellar infarction was found to be remarkably lower than that in the control group, suggesting that the occurrence of cognitive impairment after cerebellar infarction may be intimately related to the change of structural connections in the precuneus.

In addition, there were significant differences in the nodal efficiency of some brain regions between the two groups, mainly in the precuneus, cingulate gyrus and frontal-temporal cortex. The reduced efficiency of nodes reflected disruption in the integration of structural connectivity and the dissemination of efficient information in these areas, which may be attributed to the disruption of brain network connection after cerebral infarction.

In the current study, local efficiency reductions in the bilateral PCUN, PCG and DCG were found. The PCUN and cingulate gyrus are the core components of the default mode network (DMN) (44). Previous studies on Alzheimer's disease have shown that the disintegration of the DMN was related to dysfunctions of cognition, such as memory and information processing (45, 46). The PCUN has been shown to be involved in many advanced functional activities in previous studies, such as episodic memory retrieval, visuospatial imagery, self-processing, consciousness (47–49). Cunningham et al used diffusion tensor imaging (DTI) and found that there was significant structural connectivity between the PCUN and the thalamus, hippocampus and middle prefrontal cortex (50). Another study also indicated that the cerebellum, especially Crus I and Crus II, had extensive neural connections with the PCUN (51). Halko et al indicated that intermittent theta-burst stimulation of the lateral cerebellum can increases functional connectivity of the default network (52). At present, multiple studies have shown that the cerebellum contributes significantly to the default network (53–55). Furthermore, the cingulate gyrus also plays an important role in the DMN. The cingulate gyrus, especially the PCG, had a rich connection with other brain regions, such as the thalamus and basal ganglia, which was also an important part of the Papez loop (mainly composed of the hippocampus, thalamus and cingulum) (56). Previous studies also showed that there were neural circuits between the cerebellum and specific regions of the cerebrum, such as the cingulate gyrus and frontal and temporal lobes (8). There was also a close relationship between the anterior, medial and posterior cingulate gyrus (57). Kobayashi found that the cingulate gyrus plays a key role in regulating spatial cognition, working memory processing and episodic memory formation (58). Regarding the study of the cingulate gyrus, most of the current studies have considered that the anterior cingulate gyrus was related to complex physical and visceral motor functions, cognitive function, emotional processing and pain responses, the PCG was related to cognitive function, mainly visual spatial ability and memory (59, 60), and the DCG was considered to have the function of regulating attention and executive functions (61).

There was also a reduction in local efficiency in some frontal and temporal cortices, including the inferior frontal gyrus, opercular part, inferior frontal gyrus, orbital part, temporal pole, superior temporal gyrus, and temporal pole, middle temporal gyrus. A meta-analysis showed that there were extensive neural connections between the cerebellum and the cerebrum, and one importantly connected mode was the connection between the posterior cerebellum and medial prefrontal cortex and temporoparietal junction (9). Franzmeier et al reported that the frontal lobe was specifically connected to the dorsal attention network and the DMN (two core cognitive networks) and plays various functions in learning, working memory, planning, selective attention, and cognitive flexibility (62). Findings in our study support that cognitive impairment may be mainly attributed to the decreased efficiency of local connections in the brain regions involved in default networks and frontotemporal lobe after cerebellar infarction. Furthermore, previous studies have documented that there are ipsilateral and contralateral fibrous connections between the cerebellum and the brain (9, 63). Current studies have also revealed that the changes of network connections in bilateral brain areas after cerebellar infarction may be associated with ipsilateral and contralateral regulation of the cerebellum, which requires to be further explored.

At the same time, correlation analysis was performed between the differences of whole brain structural connectivity and neuropsychological scores. In terms of general cognitive assessment, we found that the MMSE scores were negatively correlated with *Lp* and positively correlated with local efficiency of some brain regions, including the right DCG, right PCG and right PCUN. We also found a significant correlations between episodic memory, executive function, visuospatial abilities and processing speed with structural network abnormalities. In particular, increased *Lp* was manifested as a significant correlation with deficits of episodic memory and executive function; reduction in *Eglob, Eloc* and the efficiency of several brain regions (right DCG, right PCG, and right PCUN) showed significant correlations with impaired executive function; the local efficiency of right DCG and right PCG showed significant correlations with impaired visuospatial abilities; disrupted brain regions of the left DCG were significantly correlated with disrupted processing speed. The correlation analysis suggested that the abnormal alterations in the right PCG, bilateral DCG, right PCUN may play a core role in the cognitive impairment following cerebellar infarctions.

The cerebellum participated in the cognitive processing process, including memory, language functions, visuospatial abilities and processing speed. The cerebellum regulated the cognitive functional regions of the brain through a wide range of neural circuits formed between the cerebrum and cerebellum. The mechanisms of cognitive dysfunction after cerebellar lesions may be by interrupting the nodal efficiency of the structural network and disrupting integration of brain networks.

## Limitations

Although we have achieved some considerable results, there are also some methodological problems in our research. First, owing to the small sample size, we have no subgroup of patients with cerebellar infarction based on the left and right location. Therefore, we will continue to recruit patients with cerebellar infarction and conduct in-depth explorations of the functional changes related to different aspects of the lesions. In addition, in our study, deterministic tractography was used to define the edge of the structural network. This analysis might have led to the loss of tracking existing fibers due to fiber crossing problems and limitations of adequately tracking long-distance fibers. An additional supplemental approach may be needed, such as probabilistic fiber tracking, to verify our results.

## Conclusions

In general, we are the first to use diffusion-weighted MRI to study the brain structural network of patients with cognitive impairment following cerebellar infarction. Our findings suggested that cognitive impairment after cerebellar infarction was associated with decreased connectivity and information transmission in some brain regions, such as bilateral PCUN, PCG, and DCG, as well as frontotemporal lobes. Among them, abnormal changes of right PCG, bilateral DCG and right PCUN might play a central role in cognitive impairment. Our findings validate the concept of cerebro-cerebellar circuits and indicate that there will be disruptions in structural brain networks after cerebellar infarction. The mechanism of cognitive impairment after cerebellar injury may be the destruction in the connection efficiency and information integration ability of brain network, suggesting that cerebellum may only participate in the integration and regulation of brain network connection, so as to make it function more effectively. The disruptions in the topological features of the structural brain networks within the cerebro-cerebellar circuits may provide a new approach to explore the pathophysiological mechanisms of cognitive impairment following cerebellar infarction.

## Data Availability

Datasets are available upon request to the authors.

## Ethics Statement

This study was carried out in accordance with the recommendations of the Ethics Committee of the Affiliated Brain Hospital of Nanjing Medical University with written informed consent from all subjects. All subjects gave written informed consent in accordance with the Declaration of Helsinki. The protocol was approved by the Ethics Committee of the Affiliated Brain Hospital of Nanjing Medical University.

## Author Contributions

JS designed the study and revised it critically for important intellectual content. DW performed the research and drafted the manuscript. QY, MY, and LF helped in data analyses. XL, CX, DZ, and MT help in clinical data collection, analyses, and made patients follow-ups.

### Conflict of Interest Statement

The authors declare that the research was conducted in the absence of any commercial or financial relationships that could be construed as a potential conflict of interest.

## Abbreviations

CCAS: Cerebellum cognitive affective syndrome
DTI: Diffusion imaging technology
MMSE: Mini-Mental Status Examination
RAVLT: Rey auditory verbal learning test
DST: Digit span test
TMT: Trail-Making Test
VFT: Verbal fluency task
BNT: Boston naming test
BBS: Berg balance scale
ICARS: International Cooperative Ataxia Rating Scale
CDT: Clock drawing test
HAMD: Hamilton Depression Scale
Eloc: Local efficiency
Eglob: Global efficiency
Cp: Clustering coefficient
γ: Normalized clustering coefficient
Lp: Shortest path length
λ: Normalized characteristic path length
σ: Small-worldness
THA: Thalamus
MTG: Middle temporal gyrus
CAU: Caudate nucleus
PoCG: Postcentral gyrus
ITG: Inferior temporal gyrus
DCG: Median cingulate and paracingulate gyri
CUN: Cuneus
SFGdor, Superior frontal gyrus: dorsolateral
CAL: Calcarine fissure and surrounding cortex
PUT, Lenticular nucleus: putamen
PCUN: Precuneuses
MOG: Middle occipital gyrus
ORBmid, Middle frontal gyrus: orbital part
PCG: Posterior cingulate gyrus
PreCG: Precentral gyrus
SOG: Superior occipital gyrus
IFGoperc, Inferior frontal gyrus: opercular part
ORBinf, Inferior frontal gyrus: orbital part
SMA: Supplementary motor area
TPOsup, Temporal pole: superior temporal gyrus
TPOmid, Temporal pole: middle temporal gyrus
DMN: Default mode network.
